# BACE1 Regulates Proliferation and Neuronal Differentiation of Newborn Cells in the Adult Hippocampus in Mice

**DOI:** 10.1523/ENEURO.0067-18.2018

**Published:** 2018-08-03

**Authors:** Zena K. Chatila, Eunhee Kim, Clara Berlé, Enjana Bylykbashi, Alexander Rompala, Mary K. Oram, Drew Gupta, Sang Su Kwak, Young Hye Kim, Doo Yeon Kim, Se Hoon Choi, Rudolph E. Tanzi

**Affiliations:** 1 Genetics and Aging Research Unit, Department of Neurology, Massachusetts General Hospital, Harvard Medical School, Charlestown, Massachusetts 02129,; 2 Biomedical Omics Group, Korea Basic Science Institute, Cheongju-si, Chungbuk 363-883, Republic of Korea

**Keywords:** Adult neurogenesis, Alzheimer’s disease, BACE1, hippocampus

## Abstract

β-Site amyloid precursor protein cleaving enzyme 1 (BACE1) is required for the production of β-amyloid (Aβ), one of the major pathogenic molecules of Alzheimer’s disease (AD), and is therefore being actively pursued as a drug target for AD. Adult hippocampal neurogenesis (AHN) is a lifelong process that is known to be important for learning and memory and may have the potential to regenerate damaged neural tissue. In this study, we examined whether BACE1 regulates AHN, which holds important implications for its suitability as a drug target in AD. Cohorts of 2-month-old wild-type (BACE1^+/+^), heterozygous, and homozygous BACE1 knockout mice (BACE1^+/–^ and BACE1^–/–^, respectively) were injected with 5-bromo-2′-deoxyuridine (BrdU) and sacrificed 1 day later to examine the impact of loss of BACE1 on neural precursor cell (NPC) proliferation in the adult brain. Parallel cohorts of mice were sacrificed 4 weeks after BrdU injection to determine the effects of BACE1 on survival and differentiation of newborn NPCs. We found that NPC proliferation was increased in BACE1^–/–^ mice compared to BACE1^+/+^ mice, while no difference was observed in NPC survival across genotypes. Differentiation of NPCs to neuronal lineage was impaired in BACE1^–/–^ mice. However, no differences were observed in astrogenesis, the proportion of immature neurons, or the production of oligodendrocytes across genotypes. Importantly, corresponding with a decrease in neuronal differentiation in the absence of a complementary increase in an alternate cell fate, BACE1^–/–^ mice were found to have a pool of undifferentiated NPCs in the hippocampus compared to BACE1^+/+^ and BACE1^+/–^ mice.

## Significance Statement

The present findings not only demonstrate that BACE1 regulates AHN, but also raise a note of caution that therapeutic inhibition of its enzymatic activity might have the potential to produce a pool of adult-generated undifferentiated cells in the hippocampus. Our data strongly suggest that the complete loss of BACE1 activity dysregulates AHN in the adult mouse hippocampus. Alternatively, partial inhibition of BACE1 activity, e.g., in BACE1^+/–^ mice, may mirror a more suitable therapeutic approach that would not impact AHN or NPC differentiation, for when BACE1 inhibition is being pursued as an effective means of lowering Aβ levels in AD.

## Introduction

β-Site amyloid precursor protein cleaving enzyme 1 (BACE1) is required for the production of β-amyloid 42 (Aβ42; Tanzi and Bertram, 2005). When produced excessively, Aβ42 aggregates to form amyloid plaques, a pathologic hallmark of Alzheimer’s disease (AD). Importantly, amyloid pathology is absent in transgenic AD mice crossed with those lacking the BACE1 gene. As a result, down-regulation of BACE1 activity is being investigated to treat or prevent AD (Vassar, 2014). However, endogenous roles of BACE1 have yet to be fully determined, and it is possible that inhibiting BACE1 may compromise neural function. It is therefore critical to understand the physiologic functions of BACE1 to fully comprehend the effects of manipulating BACE1 activity.

The human hippocampus contains neural progenitor cells (NPCs) that continue to generate substantial levels of new neurons at a steady rate well into old age, with only a modest decline (Eriksson et al., 1998; Spalding et al., 2013). This process is known as adult hippocampal neurogenesis (AHN). AHN is believed to underlie mechanisms of neural plasticity, learning, and memory, and may be critical for regeneration of damaged neural tissue (Zhao et al., 2008; Ming and Song, 2011; Aimone et al., 2014). It has been shown that AHN is altered in early stages of AD in various AD transgenic animal models (Mu and Gage, 2011).

BACE1 is known to regulate neurogenesis in early developmental stages (Hu et al., 2013). Additionally, sAPPβ, a direct product of the cleavage of the amyloid precursor protein (APP) by BACE1, also regulates proliferation and differentiation of neural precursor cells (NPCs) *in vitro* (Freude et al., 2011; Baratchi et al., 2012). Neuregulin 1, which is cleaved by BACE1, has been shown to regulate AHN (Mahar et al., 2016). However, the involvement of BACE1 in regulating AHN, which holds important implications for its suitability as a drug target in AD, has yet to be determined. In the present study, we investigated whether BACE1 plays critical roles in AHN by examining proliferation, survival, and differentiation of newborn cells in the dentate gyrus (DG) of BACE1 knockout (KO) mice. Determining whether BACE1 activity regulates AHN will help to elucidate whether therapeutically decreasing the activity of this enzyme will further impair neuronal functioning in AD.

## Materials and methods

### Animals

Heterozygous BACE1 KO mice (BACE1^+/–^, B6SJL129 × C57Bl/6 mixed background) were kindly provided by the laboratory of Dr. Mark Albers (Massachusetts General Hospital) and maintained in our facility by crossing male and female heterozygous mice. Male wild-type (BACE1^+/+^ mice), BACE1^+/–^, and homozygous BACE1 KO (BACE1^–/–^) mice were used for this study. Animals were housed with a 12-h:12-h light-dark cycle and with food and water provided *ad libitum*. All animal procedures were performed in accordance with Massachusetts General Hospital animal care committee’s regulations.

### Immunohistochemistry for BACE1

BACE1^+/+^, BACE1^+/–^, and BACE1^–/–^ mice were deeply anesthetized with isoflurane and perfused transcardially with 0.9% NaCl followed by 4% paraformaldehyde (PFA) in cold 0.1 m phosphate buffer (pH 7.4). The brains were postfixed overnight in 4% PFA and then transferred into 30% sucrose and kept there until they sank. Brains were sectioned coronally at 40 µm with a sliding freezing microtome (SM 2010R, Leica Biosystems). The brain sections were stained by rabbit anti-BACE1 (D10E5, 1:400, Cell Signaling Technology). The immunohistochemical staining was made using the avidin-biotin complex (ABC) system (Vectastain Elite, Vector Labs) and nickel-enhanced diaminobenzidine (DAB) incubation. Sections were mounted on gelatin-coated slides, air-dried, dehydrated through a graded alcohol series, cleared, and coverslipped for microscopic examination.

### 5-Bromo-2′-deoxyuridine injection and quantification and phenotype of newborn cells

5-Bromo-2′-deoxyuridine (BrdU; Sigma) was dissolved in 0.9% NaCl at a concentration of 20 mg/ml and was filtered (0.2 µm) under sterile conditions. Mice received a single i.p. injection of BrdU (100 mg/kg, Sigma) at the age of 2 months. Half of the mice in each group were sacrificed 1 d after the injection to determine progenitor proliferation. The remaining mice were sacrificed 4 weeks after the BrdU injection to determine survival and neuronal differentiation of the newborn cells.

Immunofluorescent labeling for BrdU, neuronal nuclei (NeuN), doublecortin (DCX), glial fibrillary acidic protein (GFAP), and Olig2 (O2) was performed. The antibodies used were as follows: rat anti-BrdU (1:100, Accurate Chemical & Scientific Corp.), mouse anti-NeuN (1:500, Millipore Bioscience Research Reagents), goat anti-DCX (1:200, Santa Cruz Biotechnology), rabbit anti-GFAP (1:500, Dako), and rabbit anti-O2 (1:100, Abcam). The fluorescent secondary antibodies used were donkey anti-rat IgG conjugated with Cy2; donkey anti-mouse IgG conjugated with Cy3 or Cy5; donkey anti-goat IgG conjugated with Cy3 or Cy5; donkey anti-rabbit IgG conjugated with Cy3 or Cy5 (all 1:250, Jackson ImmunoResearch).

For BrdU staining, DNA was denatured by incubating the sections for 2 h in 50% formamide/2× SSC (0.3 m NaCl and 0.03 m sodium citrate) solution at 65°C. Sections were rinsed for 15 min in 2× SSC and incubated for 30 min in 2 N HCl at 37°C. The acid was neutralized by rinsing the sections for 10 min in 0.1 m boric acid (pH 8.5) followed by several washes in Tris-buffered saline (TBS, pH 7.5). Tissues were then mounted with polyvinyl alcohol with diazabicyclo-octane (PVA-DABCO, Sigma) for viewing and imaging with a Nikon Eclipse Ti confocal laser scanning microscope (Nikon Instruments).

To estimate the total number BrdU^+^ cells, every sixth brain section was systematically selected for immunostaining, and BrdU^+^ cells in the subgranular and granular cell layers were counted. Resulting numbers were multiplied by six to obtain the estimated total number of BrdU^+^ cells per animal. For colabeling analysis of differentiated BrdU^+^ cell types with lineage-specific markers, the phenotypes of 15–20 BrdU^+^ cells per animal were determined. The total number of GFAP^+^ cells in the DG was counted in every sixth brain section, and GFAP signal intensity (integrated density) was measured by NIH ImageJ software.

### Quantification of Ki67^+^ cells

Immunofluorescent labeling for Ki67 was performed using rabbit anti-Ki67 antibodies (1:100, Abcam) and donkey anti-rabbit IgG conjugated with Cy3 (1:250, Jackson ImmunoResearch). 4′,6-Diamidino-2-phenylindole (DAPI) counterstaining was performed to measure DG volume using a NIS-Elements AR (Nikon Instruments). Every sixth brain section was systematically selected for immunostaining, and Ki67^+^ cells in the subgranular and granular cell layers were counted. The Ki67^+^ cell counts were expressed and analyzed as the number of total Ki67^+^ cells divided by the DG volume to normalize for variations across mice; BACE1^–/–^ mice showed smaller sizes of brain and DG than BACE1^+/+^ and BACE1^+/–^ mice.

### Immunoblot analysis

Hippocampal tissues were homogenized in extraction buffer containing 2% SDS and 1% Triton. 50 µg of protein were resolved on 12% Bis-Tris or 4%–12% gradient Bis/Tris gels (Life Technologies), and the proteins were transferred to nylon membranes (Bio-Rad). Immunoblot images were visualized by enhanced chemiluminescence (ECL). The images were captured by using BioMax film (Kodak) or VersaDoc imaging system (Bio-Rad). The following primary antibodies were used: full-length APP (C66, a gift from Drs. Dora M. Kovacs and Doo Yeon Kim at Massachusetts General Hospital), APP-CTFα (C66), APP-CTFβ (C66), BACE1 (D10E5, Cell Signaling Technology), and β-actin (Sigma).

### Statistics

Data are expressed as mean values ± SEM. Error bars in the figures represent SEM. Differences between groups were analyzed using ANOVA followed by a *post hoc* comparison using Tukey. Statistical analysis was performed using PRISM GraphPad statistical software. In all cases, *p* = 0.05 was considered statistically significant.

## Results

### Lack of BACE1 expression in BACE1^–/–^ mice

**Figure 1.**
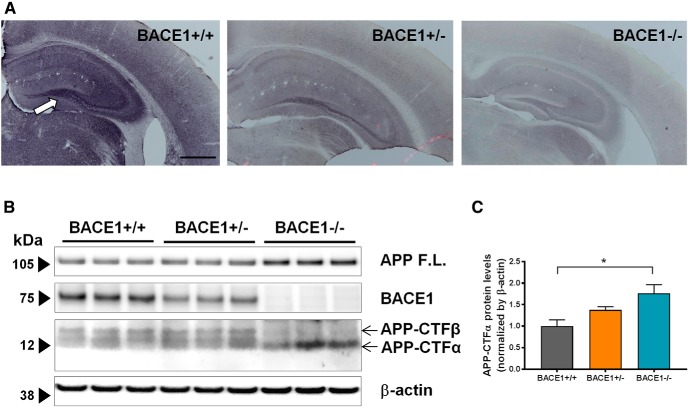
BACE1 is not expressed in BACE1^–/–^ mice. ***A***, BACE1 expression was assessed in coronal sections of 2-month-old BACE1^+/+^ (left), BACE1^+/–^ (middle), and BACE1^–/–^ (right) mice using immunohistochemistry with a commercial BACE1 antibody. BACE1 immunoreactivity is present throughout BACE1^+/+^ sections, with higher levels in the mossy fiber pathway of the hippocampus (white arrow). Scale bar, 500 µm. Conversely, there is no BACE1 immunoreactivity in BACE1^–/–^ brain sections. ***B***, BACE1 expression is attenuated in BACE1^+/–^ and abrogated in BACE1^–/–^ mice. The expression and activity of BACE1 was assessed in BACE1^+/+^, BACE1^+/–^, and BACE1^–/–^ mice with Western blot. BACE1 is expressed robustly in BACE1^+/+^ brain tissues, is weakly expressed in BACE1^+/–^ brain tissues, and is not expressed in BACE1^–/–^ brain tissues, while β-actin controls are expressed equally in all three. APP-CTFα, a product of the enzymatic cleaving of APP by α secretase, is present in tissues from all three genotypes. However, APP-CTFβ, a product of the enzymatic cleaving of APP by BACE1, is present in BACE1^+/+^ and BACE1^+/–^ tissues, but not in BACE1^–/–^ tissues. ***C***, Densitometric analysis of APP-CTFα. *F*_(2,6)_ = 6.557, *p* = 0.0309; *, *p* < 0.05.

BACE1 expression was assessed through immunohistochemistry to confirm the lack of BACE1 expression in BACE1^–/–^ mice. In BACE1^+/+^ brain tissues, diffuse BACE1 immunoreactivity was present throughout entire sections, and was higher in the mossy fiber pathway of the hippocampus (Fig. 1*A*), as previously reported (Zhao et al., 2007). In BACE1^+/–^ mice, BACE1 immunoreactivity was reduced but still present in the mossy fiber pathway, but no BACE1 immunoreactivity was observed in brain sections from BACE1^–/–^ mice.

BACE1^+/+^, BACE1^+/–^, and BACE1^–/–^ brain tissues were also assessed for BACE1 expression through Western blot analysis (Fig. 1*B*). As expected, BACE1 was robustly expressed in BACE1^+/+^ brain tissues, weakly expressed in BACE1^+/–^ tissues, and not expressed in BACE1^–/–^ tissues. To assess BACE1 activity, brain tissues were also examined for the C-terminal APP fragment β subunit (APP-CTFβ), a product of its enzymatic cleaving of APP. We confirmed that APP-CTFβ levels are absent in hippocampal brain tissues of BACE1^–/–^ mice. The level of full-length APP (APP F.L.) was increased in BACE1^–/–^ mice, as previously reported (Pastorino et al., 2004). The level of APP-CTFα is expected to increase by compensation of α-secretase activity in the absence of BACE1, and we observed that its level was increased in BACE1^–/–^ mice compared to BACE1^+/+^ mice (Fig. 1*C*).

### Lack of BACE1 increases NPC proliferation without affecting NPC survival

**Figure 2.**
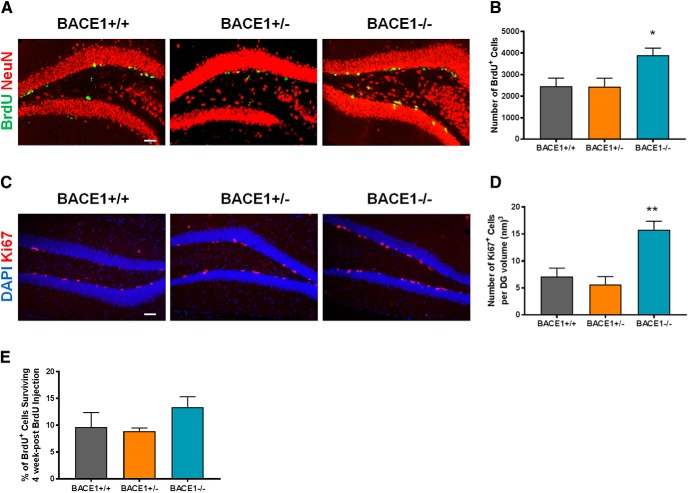
Loss of BACE1 increases NPC proliferation without affecting NPC survival. ***A***, Photomicrographs of BrdU^+^ cells 1 d after BrdU injection in the DG of BACE1^+/+^, BACE1^+/–^, and BACE1^–/–^ mice. Green or yellow, BrdU^+^ cells; red, NeuN^+^ cells. Scale bar: 50 µm. ***B***, Quantification of BrdU^+^ cells 1 d post-BrdU injection (mean ± SEM; *n* = 6 in BACE1^+/+^, 6 in BACE1^+/–^, and 5 in BACE1^–/–^ mice; *F*_(2,14)_ = 4.629, *p* = 0.0286); *, *p* < 0.05. ***C***, Photomicrographs of Ki67^+^ cells in the DG of BACE1^+/+^, BACE1^+/–^, and BACE1^–/–^ mice. Red, Ki67^+^ cells; blue, DAPI^+^ cells. Scale bar: 50 µm. ***D***, Quantification of Ki67^+^ cells (mean ± SEM; *n* = 5 in each group; *F*_(2,12)_ = 15.52, *p* = 0.0012); **, *p* < 0.01. ***E***, To provide a measure of cell survival during the 4-week post-BrdU time period, the number of BrdU^+^ cells at the 4-week post-BrdU time point was expressed as a percentage of the number present at the 1-d post-BrdU time point (mean ± SEM; *n* = 6 in BACE1^+/+^, 8 in BACE1^+/–^, and 5 in BACE1^–/–^ mice; *F*_(2,16)_ = 1.654, *p* = 0.2224).

To examine whether BACE1 affects the proliferation of NPCs, 2-month-old BACE1^+/+^, BACE1^+/–^, and BACE1^–/–^ mice were injected with BrdU and sacrificed 1 d after injection. The total number of BrdU^+^ cells was subsequently determined for each animal. We observed a marked increase in the number of BrdU^+^ cells in BACE1^–/–^ mice compared to BACE1^+/+^ and BACE1^+/–^ mice (Fig. 2*A*,*B*). In addition to the BrdU analysis, we also quantified the number of cells expressing Ki67, a cellular marker for proliferation. As observed with BrdU, the number of Ki67^+^ cells was significantly higher in 2-month-old BACE1^–/–^ mice compared to age-matched BACE1^+/+^ and BACE1^+/–^ mice (Fig. 2*C*,*D*). The effects of the partial loss of BACE1 activity in BACE1^+/–^ mice did not increase NPC proliferation in both BrdU and Ki67 analyses. These results suggest that complete loss of BACE1 increased NPC proliferation in DG of adult mice.

To examine whether BACE1 activity affects survival of NPCs, parallel cohorts of mice were sacrificed 4 weeks after BrdU injection. The total number of BrdU^+^ cells was determined for each animal. A measure of cell survival was provided by expressing the number of BrdU^+^ cells at 4 weeks after BrdU injection as a percentage of the overall average number of BrdU^+^ cells at 1 d postinjection for each respective genotype. We failed to observe any differences in cell survival across the three genotypes (Fig. 2*E*).

### Lack of BACE1 impairs neuronal fate differentiation of newborn cells

**Figure 3.**
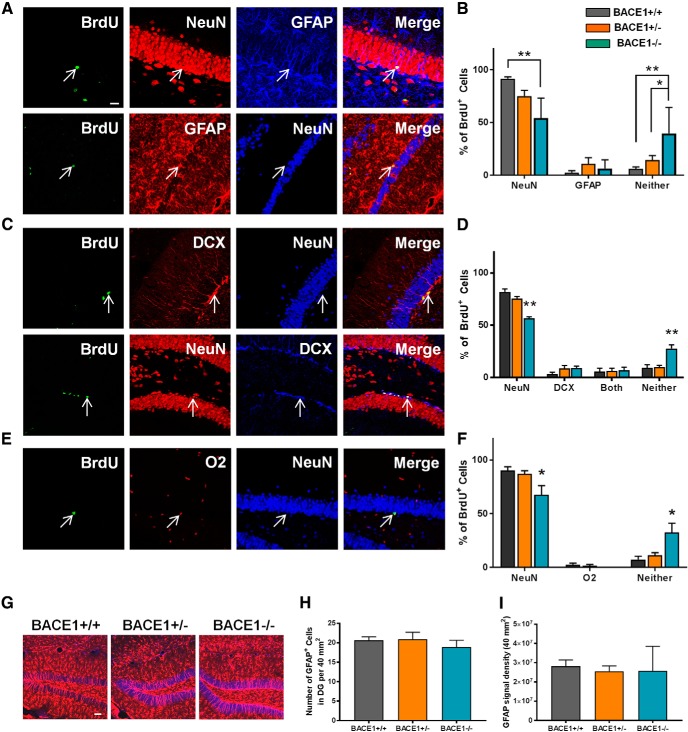
Loss of BACE1 alters neuronal fate differentiation of NPCs. ***A***, Representative confocal images of BrdU^+^ cells colabeled with NeuN (upper panels) or GFAP (lower panels). Arrows indicate the position of BrdU^+^ cells. Scale bar: 20 µm. ***B***, Percentage of BrdU^+^ cells colabeled with NeuN or GFAP, or neither of them (mean ± SEM; *n* = 6 in BACE1^+/+^, 8 in BACE1^+/–^, and 5 in BACE1^–/–^ mice). For NeuN, *F*_(2,16)_ = 9.501, *p* = 0.0019. For GFAP, *F*_(2,16)_ = 0.786, *p* = 0.4725. For neither, *F*_(2,16)_ = 7.551, *p* = 0.0049; *, *p* < 0.05; **, *p* < 0.01. ***C***, Representative confocal images of BrdU^+^ cells colabeled with DCX but not NeuN (upper panels), or with DCX and NeuN (lower panels). ***D***, Percentage of BrdU^+^ cells colabeled with NeuN or DCX, both, or neither of them (mean ± SEM; *n* = 6 in BACE1^+/+^, 8 in BACE1^+/–^, and 5 in BACE1^–/–^ mice). For NeuN, *F*_(2,16)_ = 28.34, *p* < 0.0001. For DCX, *F*_(2,16)_ = 1.566, *p* = 0.2392. For both, *F*_(2,16)_ = 0.03229, *p* = 0.9863. For neither, *F*_(2,16)_ = 13.63, *p* = 0.0004; **, *p* < 0.01. ***E***, Representative confocal images of BrdU^+^ cells colabeled with O2. ***F***, Percentage of BrdU^+^ cells colabeled with NeuN or O2, or neither of them (mean ± SEM; *n* = 4 in BACE1^+/+^, 6 in BACE1^+/–^, and 4 in BACE1^–/–^ mice). For NeuN, *F*_(2,11)_ = 5.607, *p* = 0.0210. For O2, *F*_(2,11)_ = 1.235, *p* = 0.3283. For neither, *F*_(2,11)_ = 7.3, *p* = 0.0096; *, *p* < 0.05. ***G***, Representative confocal images of GFAP^+^ astrocytes in the DG of BACE1^+/+^, BACE1^+/–^, and BACE1^–/–^ mice. Red, GFAP^+^ cells; blue, NeuN^+^ cells. Scale bar: 50 µm. ***H***, Quantification of GFAP^+^ cells (mean ± SEM; *n* = 5 per group; *F*_(2,12)_ = 0.5162, *p* = 0.6095). ***I***, GFAP signal intensity (mean ± SEM; *n* = 5 per group; *F*_(2,12)_ = 0.0344, *p* = 0.9662).

NPCs that reach maturity are fated to become either granule neurons or glial cells. To determine the effect of BACE1 activity on the differentiation of newborn NPCs, we performed triple-labeling studies using anti-BrdU antibodies combined with antibodies specific for neuronal, glial, or oligodendrocytic lineages 4 weeks post BrdU injection (Fig. 3*A–F*). We examined expression of the following markers: NeuN, a mature neuronal marker; glial fibrillary acidic protein (GFAP), an astrocyte marker; doublecortin (DCX), an immature neuronal marker; and O2, an oligodendrocyte-specific marker.

On examination of BrdU^+^ cells colabeled with either NeuN or GFAP, the proportion of BrdU^+^ cells expressing NeuN was decreased in BACE1^–/–^ compared to BACE1^+/+^ mice (Fig. 3*A*,*B*). This finding demonstrates that eliminating BACE1 activity impairs neuronal differentiation of newborn cells in the adult DG. No differences were observed in the proportion of BrdU^+^ cells colabeled with GFAP across the three genotypes, indicating that BACE1 does not affect the differentiation of NPCs into astrocytic lineages in the adult brain. Importantly, the proportion of BrdU^+^ cells that were labeled with neither NeuN nor GFAP was higher in BACE1^–/–^ mice than in either BACE1^+/+^ or BACE1^+/–^ mice, between which no differences were observed.

It is possible that this pool of seemingly undifferentiated BrdU^+^ cells in BACE1^–/–^ mice is partly comprised of immature neurons, and that some consequences of the BACE1^–/–^ genotype include a delay of neuronal maturation. Neurons developing in BACE1^–/–^ mice may be stalled at immature stages and fail to reach a fully mature phenotype; maturation may occur at different rates in the DG of BACE1^–/–^ mice. As a result, BrdU^+^ cells were additionally assessed for colabeling with NeuN or DCX to determine if BACE1 regulates the rate of neuronal maturation (Fig. 3*C*,*D*). This experiment also aimed to better characterize the pool of BrdU^+^ cells in BACE1^–/–^ mice that adopted neither a mature neuronal nor an astrocytic fate. Again, the proportion of BrdU^+^ cells expressing NeuN was decreased in BACE1^–/–^ compared to BACE1^+/+^ mice, confirming that a lack of BACE1 activity impaired neuronal differentiation of newborn NPCs. No differences were observed in the proportion of BrdU^+^ cells colabeled with DCX across the three genotypes, indicating that BACE1 does not affect the rate of maturation of newborn neurons. Importantly, an increased proportion of BrdU^+^ cells that were neither labeled with NeuN nor with DCX, and that therefore did not adopt a neural fate, was observed in BACE1^–/–^ compared to BACE1^+/+^ and BACE1^+/–^ mice.

BACE1 regulates the process of myelination and myelin sheath thickness in the central nerves. The genetic deletion of BACE1 delays the process of neuregulin-dependent myelination and reduces myelin thickness (Hu et al., 2006; Willem et al., 2006). Thus, the hypomyelination in BACE1^–/–^ mice may alter AHN processes in these mice; it is also possible that this pool of apparently undifferentiated cells in BACE1^–/–^ mice may be oligodendrocytes that are being overproduced as a compensatory mechanism. To determine if BACE1 regulates the production of oligodendrocytes, BrdU^+^ cells were additionally assessed for colabeling with NeuN or O2. The proportion of BrdU^+^ cells expressing NeuN was decreased in BACE1^–/–^ compared to BACE1^+/+^ mice, yet again confirming that a lack of BACE1 activity impaired neuronal differentiation of newborn NPCs (Fig. 3*E*,*F*). However, no differences were observed in the proportion of BrdU^+^ cells colabeled with O2 across the three genotypes, indicating that BACE1 does not affect the production of oligodendrocytes. Importantly, an increased proportion of BrdU^+^ cells that were labeled with neither NeuN nor O2 was observed in BACE1^–/–^ compared to both BACE1^+/+^ and BACE1^+/–^ mice.

The effect of decreased BACE1 activity on mature neuronal fate in BACE1^+/–^ mice is less clear. In the immunostaining assay with BrdU, NeuN, and GFAP, no differences were observed in the proportion of BrdU^+^ cells colabeled with NeuN between BACE1^+/–^ and either BACE1^+/+^ or BACE1^–/–^ mice (Fig. 3*B*). However, in the separate immunostaining with BrdU, NeuN, and either DCX or O2, we did observe a difference between BACE1^+/–^ and BACE1^–/–^ mice but not between BACE1^+/–^ and BACE1^+/+^ mice (Fig. 3*D*,*F*).

## Discussion

The present study investigated whether BACE1 regulates AHN in BACE1-null mice. We found that complete loss of BACE1 increased NPC proliferation in the DG of adult mice. Our results are in contrast with a previous report showing that inhibition of BACE1 activity decreases proliferation of cultured human embryonic stem cells (hESCs; Porayette et al., 2009). It is possible that the effects of BACE1 on NPC proliferation are age dependent and/or non–cell autonomous in mice, which would account for the differences between the present results and those from the previous study. It is also possible that the increased NPC proliferation in our BACE1^–/–^ mice is a result of compensation of soluble APPα (sAPPα), which has been shown to stimulate NPC proliferation (Baratchi et al., 2012), and is elevated when BACE1 is absent or inhibited (Nishitomi et al., 2006). In any event, the findings from this study suggest that BACE1 inhibition during adulthood increases NPC proliferation.

We also demonstrated that the absence of BACE1 activity produced a pool of NPCs that adopts neither a neuronal, an astrocytic, nor an oligodendrocytic fate, and thus appears to be undifferentiated. Further studies are required to elucidate mechanisms by which AHN is altered in BACE1^–/–^ mice. BACE1-null mice in early developmental stages have been found to have decreased hippocampal neurogenesis and a corresponding increase in astrogenesis compared to wild-type counterparts, due to BACE1 cleavage of jagged-1 and consequent changes in the Notch signaling pathway in BACE1-null mice (Hu et al., 2013). Studies have also shown that sAPPβ induces differentiation of human embryonic stem cells toward a neural fate (Freude et al., 2011). Thus, the absence of sAPPβ in BACE1^–/–^ mice may alter adult neuronal differentiation. However, other studies conversely demonstrated that downregulation of BACE1 with siRNA is associated with an increase in several neurogenesis markers at the RNA and protein levels, suggesting that decreased BACE1 activity increases neurogenesis (Modarresi et al., 2011). It has also been shown that sAPPβ promotes astrogenesis in cultured hippocampal NPCs, suggesting that BACE1 enzymatic activity promotes astrogenesis over neurogenesis (Baratchi et al., 2012). The present finding that astrogenesis was unaffected by BACE1 activity reflects the possibility that the regulatory effects of BACE1 on astrogenesis are specific to the developmental stage of the animal, and that the role of the Notch signaling pathway in promoting astrogenesis is not maintained in adulthood. Astrogliosis has been shown to be affected by the loss of BACE1 in early developmental stages (Hu et al., 2013). However, no differences were observed in the number of GFAP^+^ cells and intensity of GFAP signal in the DG across our adult BACE1^+/+^, BACE1^+/–^, and BACE1^–/–^ mice at 2 months of age (Fig. 3*G–I*).

Our studies thus raise the concerning possibility of creating a population of undifferentiated cells, in a stem cell–like state, for an extended period time in the brains of AD patients being treated with BACE inhibitors. The resulting delay in neuronal differentiation might then lead to decreased myelination as has been observed in BACE1^–/–^ mice (Hu et al., 2006). These undifferentiated cells may be also impaired in their ability to integrate into functional circuitry, leading to the formation of aberrant patterns of connectivity that might be associated with the memory deficits and seizures observed in BACE1^–/–^ mice (Kandalepas and Vassar, 2014). Future studies will be necessary to identify these undifferentiated cells and to investigate the roles of BACE1 on AHN in conditional BACE1 KO mice. Studies may also be performed using BACE1 inhibitors in aged mice to produce insights that are more applicable to AD research.

Notably, the Merck phase 3 clinical trial of the BACE1 inhibitor, verubecestat, was terminated based on the observation of numerous adverse events including falls, weight loss, sleep disturbance, change in hair color, and suicidal ideation. These findings argue against robust inhibition of BACE1. Our findings showing that completely blocking BACE1 activity impaired AHN strongly argue against a therapeutic strategy for AD that involves robustly inhibiting BACE1. However, since AHN processes were not altered in BACE1^+/–^ mice versus BACE1^+/+^ mice, a therapeutic strategy of partial inhibition of BACE1 may still warrant further study.

## Synthesis

Reviewing Editor: Dritan Agalliu, Columbia University Medical Center Decisions are customarily a result of the Reviewing Editor and the peer reviewers coming together and discussing their recommendations until a consensus is reached. When revisions are invited, a fact-based synthesis statement explaining their decision and outlining what is needed to prepare a revision will be listed below. The following reviewer(s) agreed to reveal their identity: Robert Vassar.

The manuscript entitled “BACE1 regulates proliferation and neuronal differentiation of newborn cells in the adult hippocampus in mice” examines the role of BACE1 in the adult neurogenesis. The authors assess the effects of BACE1 inhibition on neural progenitor cells (NPC) and their differentiation in adult mice which is an important question to be considered in the current BACE inhibitors trials. Despite of no change in NPC proliferation 24 hours after BrdU injection, the authors found a significant reduction in neurogenesis (detected by co-expression of BrdU+ and the neuron marker NeuN+) and an increase of an undifferentiated pool of cells that do not express NeuN, GFAP (astrocyte marker) nor O2 (oligodendryocyte marker) in BACE1-/- mice.

The reviewers have the following concerns about the study:

1) The authors used 2-month old mice as adults. However, mice are still rapidly developing at this age [1], especially knowing BACE1-/- mice are underweight (Dominguez et al., 2002), which could suggest slower developmental growth. It is therefore an over-interpretation to imply that the current data is relevant to much more aged AD patients that the authors have mentioned multiple times throughout the manuscript. A more appropriate way to model BACE1-inhibition related effects in AD patients will be to pharmaceutically inhibit BACE1 or conditionally knockout BACE1 in aged mice.

2) Normalization of BrdU+ cells to Dentate Gyrus (DG) volume will be a better way instead of normalization to number of (DG) sections. As reported in Hu 2013, there's a decrease of NeuN+ cells in the DG of BACE1-/- mice compared to age-matched BACE1+/+ mice. They are also likely to have thinner GCL, which is not considered in the current normalizing method used in the manuscript.

3) 1. In Fig. 2 the authors should use an additional marker to label NPCs. Currently, all BrdU+ cells are considered NPCs, but this may not always be the case (eg. BrdU+ cells in the hilus).

4) 2. The authors conclude that BACE-/- mice have a pool of undifferentiated BrdU+ cells at 4 weeks post-injection. However, they do not co-stain for markers of undifferentiated cells (see previous point).

5) The level of APP CTF-α is expected to increase by compensation of α-secretase activity in the absence of BACE1. But it seems that the level of APP CTF-α actually dropped in BACE1-/- mice in Figure 1E. Please verify that the image is representative. I recommend a densitometry quantification of the blot images.

6) In contrast to Hu et al., 2013 who showed astrogliosis and astrogenesis in 1 month old BACE1-/- mice, the authors observed no differences in astrogenesis but a change in the undifferentiated pool of NPC in BACE1-/- mice at 2 months old. I wonder if the authors observed changes in astrogliosis in the 2-month old BACE1-/- mice?

7) The authors claimed that their results are in contrast to Baratchi et al. 2012 (Discussion line 4), but Baratchi et al did not directly investigate the effects of BACE1 inhibition on NPC proliferation. The fact that there is no change in the numbers of NPC (Fig. 2B) in this study could be a result of compensation of sAPPα, which also stimulates NPC proliferation as shown by Baratchi et al., 2012 and is elevated when BACE1 is absent/inhibited (Nishitomi et al., 2006).

8) The authors should carefully review their statistical reporting: how many mice, slices, cells were used in each experimental group? Please report N and whether it refers to mice, cells or slices for each group. Also report the statistical test used next to the p-value. In addition, report the actual p value for every comparison (eg. instead of p<0.05) for each comparison and include the individual points for each bar graph.

9) ANOVA gives P value for all groups in the test. Please specify what post-hoc test used for pair-wise comparison, for example, between BACE1+/+ and BACE1-/- in Figure 2B.

MINOR:

1) Please fill in [authors University] under Animal section in the Material and Methods.

2) Immunohistochemistry. Only BACE1+/+ and BACE1-/- mice were specifically mentioned, how about BACE1+/-?
